# Splenic Infarctions and Sepsis Secondary to Ceftriaxone-Associated Acute Pancreatitis: A Case of Interdisciplinary Collaboration

**DOI:** 10.7759/cureus.75592

**Published:** 2024-12-12

**Authors:** Daniel A Rabin, Max J Spiro, Connor Rachford, Timothy Fahey, John Farrell

**Affiliations:** 1 Department of Plastic Surgery, University of Illinois at Chicago, Peoria, USA; 2 Department of Pediatrics, University of Illinois at Chicago, Peoria, USA; 3 Department of Radiology, University of Illinois at Chicago, Peoria, USA; 4 Department of Medicine, Division of Infectious Disease, University of Illinois at Chicago, Peoria, USA

**Keywords:** ceftriaxone-induced pancreatitis, drug side effect, endocarditis, sepsis, splenic infarct

## Abstract

The spleen plays a crucial role in filtering aging blood cells and defending against encapsulated microorganisms. While not essential for survival, splenic dysfunction can lead to severe complications, including organ failure, infection, and death. This case study examines a rare presentation of drug-induced splenic septic thrombophlebitis secondary to pancreatitis caused by an adverse reaction to ceftriaxone. While pancreatitis is a known, common cause of splenic infarction and sepsis, there have only been two reported cases of ceftriaxone-induced pancreatitis in the past decade.

This case follows a 26-year-old female with a history of congenital truncus arteriosus, who initially presented with *Streptococcus mitis* bacteremia secondary to prosthetic heart-valve endocarditis and was treated with ceftriaxone. On day 21 of ceftriaxone treatment, the patient returned with septic splenic thrombophlebitis secondary to acute pancreatitis. The patient clinically improved and showed a decrease in inflammatory markers following the discontinuation of ceftriaxone.

These findings aim to present a rare case of ceftriaxone-induced pancreatitis. The successful diagnosis and conservative management of our patient underscore the critical importance of interdisciplinary collaboration in the management of complex cases, ultimately avoiding unnecessary surgical interventions.

## Introduction

The spleen plays a crucial role in filtering aging blood cells and defending against encapsulated microorganisms [1]. While not essential for survival, splenic dysfunction can lead to severe complications, including organ failure, infection, and death. Splenic infarction, a rare condition occurring in approximately 0.016% of hospital admissions, results from disrupted or insufficient blood supply to the spleen, leading to tissue necrosis. This interruption can involve either the afferent splenic artery or the efferent splenic vein. The primary causes of splenic infarction are thromboembolic disease and infiltrative hematologic disorders, while pancreatitis is a rare but recognized contributor [2].

This report presents a case of ceftriaxone-induced pancreatitis that led to splenic septic thrombophlebitis, initially attributed to *Streptococcus mitis* endocarditis associated with a prosthetic valve. Positron emission tomography (PET) was pivotal in identifying the splenic infection and diagnosing the pancreatitis, which was the underlying cause of splenic vein obstruction. While alcohol and gallstones account for over 90% of acute pancreatitis cases, drug-induced pancreatitis is increasingly recognized, contributing to 0.1%-2% of cases [1]. This case highlights the need for awareness of rare drug reactions and the importance of interdisciplinary collaboration in accurately diagnosing splenic thrombophlebitis, ultimately avoiding unnecessary surgical interventions in at-risk patients.

## Case presentation

This is a case of a 26-year-old female patient with a complex cardiac history, including congenital truncus arteriosus status post-repair at three weeks of age and two subsequent mitral and aortic valve replacements at ages 12 and 16, respectively. The patient initially presented with acute onset of epigastric abdominal pain, fever, and intermittent nausea without emesis or diarrhea. Blood cultures grew *Streptococcus* mitis in both sets, and both PET imaging and transthoracic echocardiography demonstrated evidence of an infectious vegetation on the prosthetic pulmonic valve, confirming clinical suspicion of prosthetic valve endocarditis, alongside a presumed aseptic splenic infarction. She was treated in accordance with guideline-directed intravenous (IV) antibiotics, and after negative serial blood cultures, a peripheral intravenous central catheter (PICC) was placed, and our patient was discharged home to complete a six-week course of intravenous ceftriaxone.

**Figure 1:**
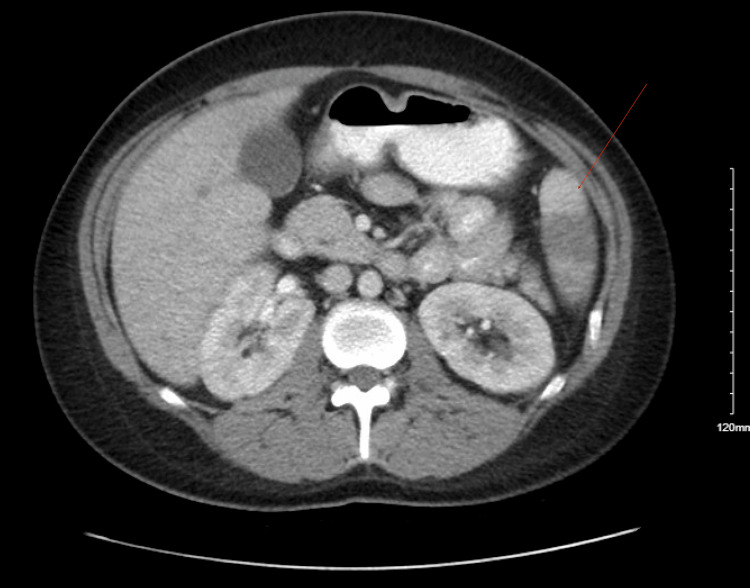
CT of the abdomen performed in the emergency department demonstrating multiple wedge-shaped non-enhancing regions off the radius for pain consistent with infarcts of the spleen.

However, the patient returned to the hospital one week later with recurrent severe epigastric pain, left upper quadrant abdominal pain, and emesis. Physical examination was remarkable for abdominal distention and left upper quadrant tenderness to palpation without rebound or guarding but was otherwise afebrile and hemodynamically stable. CT scan in the emergency department revealed acute left lower lobe subsegmental pulmonary embolism (PE) and multiple wedge-shaped non-enhancing regions consistent with infarcts of the spleen, which were presumed to be secondary to streptococcal endocarditis from the prior admission (Figure 1). Additionally, C-reactive protein (CRP) levels rose from 5.32 mg/dL to 19.56 mg/dL between previous discharge and current admission, and white blood cell (WBC) count increased from 13.70 to 16.70 cells/mm³ over the same time frame (Table 1). As a result, the patient was readmitted to the hospital for further workup.

**Table 1:** C-reactive protein (CRP) and white blood cell (WBC) count trends throughout hospital course.

	Discharge	Readmission	After ceftriaxone discontinued (day 5)	Reference range
CRP	5.32 mg/dL	19.56 mg/dL	2.84 mg/dL	<0.50 mg/dL
WBC	13.70 cells/mm^3^	16.70 cells/mm^3^	8.26 cells/mm³	4.0–12.0 cells/mm^3^

**Figure 2:**
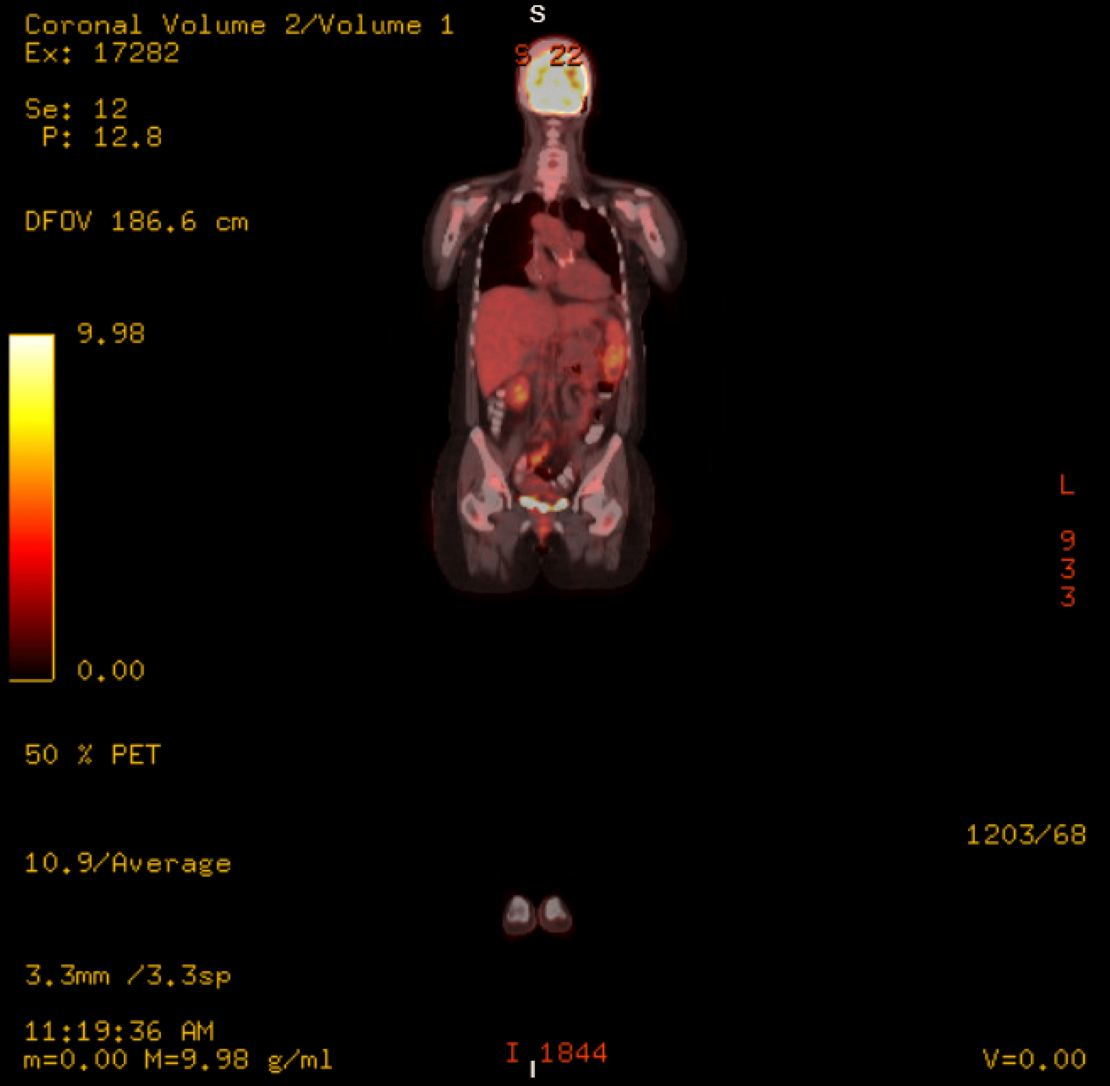
Positron emission tomography-computed tomography demonstrating a focus of fluorodeoxyglucose (FDG) uptake at the pancreatic tail associated with hypoattenuation of the parenchyma and intense focal FDG uptake at the splenic hilum in the region of the splenic vein.

**Figure 3:**
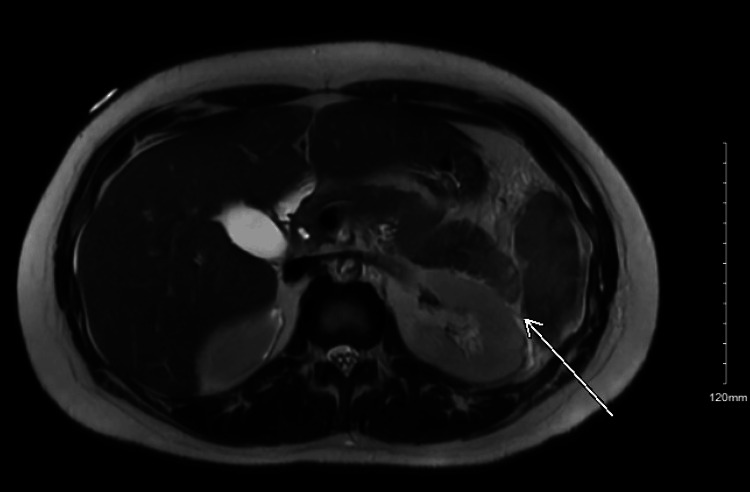
MRI confirming focal acute interstitial edematous pancreatitis.

Upon admission, the patient was switched from apixaban to warfarin due to anticoagulation failure. A second PET-CT was obtained, which demonstrated a focus of fluorodeoxyglucose (FDG) uptake at the pancreatic tail associated with hypoattenuation of the parenchyma and intense focal FDG uptake at the splenic hilum in the region of the splenic vein (Figure 2). These results raised suspicion for splenic vein septic thrombophlebitis. An MRI later confirmed focal acute interstitial edematous pancreatitis (Figure 3).

Further investigation and interdisciplinary discussions identified a link between ceftriaxone and pancreatitis: ceftriaxone can cause an accumulation of biliary sludge, leading to serious complications such as cholecystitis and pancreatitis [3]. Ceftriaxone was discontinued, and IV penicillin was initiated. Following this change, the patient’s CRP decreased from 19.56 to 2.84 mg/dL, and WBC dropped from 16.79 to 8.26 cells/mm³ (Table 1). These improvements in inflammatory markers corresponded with overall clinical improvement, and, ultimately, splenectomy was deemed unnecessary, as the patient responded remarkably well to supportive medical management.

## Discussion

Ceftriaxone-induced pancreatitis is a rare cause of drug-induced acute pancreatitis. In vitro studies have found that ceftriaxone can bind with calcium to form biliary sludge. Ceftriaxone possesses a high calcium-binding affinity. In one study, the formation constant for a calcium-ceftriaxone salt was 157.3 L/mol, with a stoichiometry of the salt at 1:1. The study found that at a dose of greater than or equal to 2g, the saturation index would surpass the metastable limit, and precipitation of ceftriaxone could occur. The study goes on to propose that, with the ceftriaxone sludge being produced, biliary “pseudolithiasis” may occur, which can then cause acute pancreatitis [4].

To better understand ceftriaxone-induced pancreatitis, we performed a literature review for case reports from the past decade (2012 to June 2022). A search of PubMed found only two case reports of ceftriaxone-induced pancreatitis during this timeframe. One report described a 70-year-old man who presented with a fever and cough for several days. He was treated with 2.0 g IV ceftriaxone for 14 days. On day 14 of admission, the patient reported a sudden onset of dyspepsia, nausea, vomiting, and epigastric pain. Ultrasound of the abdomen revealed mild thickening of the gallbladder without gallstones. A diagnosis of ceftriaxone-induced acute biliary pancreatitis was made. The patient was treated conservatively and was ultimately discharged [5].

A second case report found a nine-year-old who was treated with ceftriaxone following an appendectomy and subsequently developed pancreatitis on day 19 of ceftriaxone administration. Ultrasound revealed a gallbladder with multiple lithiasis but no main biliary duct dilation. The patient also had increased amylase and lipase levels of 1,436 and 1,046 U/L, respectively. The patient was ultimately discharged from the hospital on day 52 of admission [3].

In a similar fashion to the two cases described above, our patient presented with acute epigastric pain on day 21 of ceftriaxone administration. Using PET/CT and MRI imaging, an edematous appearing pancreatic tail with associated T2 hyperintensity was discovered. There were peripancreatic inflammatory changes and adjacent peripancreatic fat stranding. Additionally, no radiological change was noted in the biliary tree. These findings, along with the literature review, allowed us to confidently predict that the patient’s splenic infarcts were secondary to pancreatitis induced by ceftriaxone. Our prediction was further solidified when the patient’s inflammatory markers showed a downtrend following the discontinuation of ceftriaxone.

It is important to acknowledge the limitations of this case study. As a single case report, the findings may not be generalizable to broader patient populations or varying clinical scenarios, limiting the applicability of the results. Additionally, individual responses to medications can exhibit significant variability, complicating the establishment of a direct causal relationship between ceftriaxone and pancreatitis in other patients, which is ultimately a diagnosis of exclusion based on failure to detect an infectious etiology. Furthermore, the presence of potential confounding factors, such as underlying medical conditions or concomitant medications, may have contributed to the patient's clinical presentation, making it challenging to isolate the effects of ceftriaxone as the sole precipitant of pancreatitis. These limitations underscore the need for further research to clarify the implications of drug-induced pancreatitis in diverse patient cohorts.

Despite these limitations, this case underscores the critical importance of interdisciplinary collaboration in the clinical setting. The joint efforts of infectious disease specialists, radiologists, and surgeons were essential in identifying the unique link between ceftriaxone and the patient's clinical presentation of pancreatitis and subsequent splenic infarction. Through comprehensive diagnostic imaging and thorough discussions among specialists, the team was able to arrive at an accurate diagnosis, which facilitated appropriate medical management and long-term treatment decisions. This collaborative approach not only ensured effective management of the patient’s condition but also contributed valuable insights into the potential adverse effects of ceftriaxone, reinforcing the need for vigilance in monitoring drug reactions. Others can take away from this case the necessity of considering rare drug reactions in differential diagnoses, particularly in patients with complex medical histories. Additionally, this case serves as a reminder to clinicians of the importance of prompt recognition of drug-related adverse effects for a positive outcome for impacted patients.

## Conclusions

This report details a rare case of splenic infarction secondary to acute pancreatitis induced by an adverse drug reaction to appropriate antimicrobial therapy with intravenous ceftriaxone for treating *Streptococcus mitis* prosthetic valve endocarditis. A literature review identified two similar cases of pancreatitis secondary to ceftriaxone, but neither patient developed splenic arterial thromboemboli. The successful diagnosis and conservative management of our patient underscore the critical importance of interdisciplinary collaboration in the management of complex cases, ultimately avoiding unnecessary surgical interventions.

## References

[1] Chapman J, Helm TA, Kahwaji CI (2024). Splenic Infarcts. https://www.ncbi.nlm.nih.gov/books/NBK430902/.

[2] Schattner A, Adi M, Kitroser E, Klepfish A (2015). Acute splenic infarction at an academic general hospital over 10 years: presentation, etiology, and outcome. Medicine (Baltimore).

[3] Shiffman ML, Keith FB, Moore EW (1990). Pathogenesis of ceftriaxone-associated biliary sludge. In vitro studies of calcium-ceftriaxone binding and solubility. Gastroenterology.

[4] Balani AR, Grendell JH (2008). Drug-induced pancreatitis : incidence, management and prevention. Drug Saf.

[5] Sienra MC, Pereira Núñez D, Pacheco H, Juambeltz C (2020). Complications of ceftriaxone-associated biliary pseudolithiasis and neprolithiasis: a case report. Cir Pediatr.

